# The Relationships between Gender, Life Satisfaction, Loneliness and Problematic Internet Use during COVID-19: Does the Lockdown Matter?

**DOI:** 10.3390/ijerph19031325

**Published:** 2022-01-25

**Authors:** Jensen Deutrom, Vasilis Katos, Mohamed Basel Al-Mourad, Raian Ali

**Affiliations:** 1 Faculty of Science and Technology, Bournemouth University, Bournemouth BH12 5BB, UK; vkatos@bournemouth.ac.uk; 2 College of Technological Innovation, Zayed University, Dubai, United Arab Emirates; basel.almourad@zu.ac.ae; 3 College of Science and Engineering, Hamad Bin Khalifa University, Doha, Qatar

**Keywords:** problematic internet use, lockdown, working from home, life satisfaction, loneliness

## Abstract

In response to the COVID-19 pandemic, many governments have attempted to reduce virus transmission by implementing lockdown procedures, leading to increased social isolation and a new reliance on technology and the internet for work and social communication. We examined people’s experiences working from home in the UK to identify risk factors of problematic internet use during the first lockdown period, specifically looking at life satisfaction, loneliness, and gender. A total of 299 adults completed the Problematic Internet Use Questionnaire-Short-Form-6, UCLA-3 Item Loneliness Scale, and Satisfaction with Life Scale online. Through structural equation modelling, we found that loneliness positively predicted problematic internet use while gender had no effect. Life satisfaction and age positively predicted loneliness but had no direct effect on problematic internet use, suggesting loneliness fully mediated their relationship with problematic internet use. Our study serves as a benchmark study of problematic internet use among those working from home during lockdown conditions, which may be utilized by future researchers exploring longitudinal patterns post-pandemic.

## 1. Introduction

The UK is one of the countries which has been most severely affected by the COVID-19 pandemic (with the fifth highest total number of cases in the world), but also from secondary consequences such as some of the largest and most restrictive mass quarantines which have been implemented repeatedly [1,2,3]. According to the Office for National Statistics [4] nearly half of all employed people worked from home (WFH) during the first periods of lockdown, 86% of whom declaring that this was a result of the pandemic.

The restrictions brought about by the pandemic have changed our relationship with technology, with many people relying on technology and the internet for core aspects of life including work, communication, and entertainment [5,6]. While this increased use of the internet may be beneficial by helping people adapt to restrictions imposed by lockdown, this increased reliance may also foster addictive and problematic behavior [7]. Mental health and wellbeing have reportedly worsened since the pandemic, predominantly driven by the social isolation [8]. According to the ONS, 7.4 million people (almost a third of those who were asked) reported that loneliness during the first month of lockdown had negatively affected their wellbeing [9]. Additionally, research from early in the pandemic found that people felt lower life satisfaction overall, with individuals WFH reporting lower life satisfaction than those in the office but higher life satisfaction than the individuals who completely stopped to work [10]. While individuals WFH may not have suffered from financial instability to the same degree as those who were dismissed or furloughed, there is still evidence of negative impacts such as challenges linked to communicating via technology with colleagues, increased stress, reduced social contact, burnout and dilemmas with balancing life with work [11,12,13].

It is important to point out that although WFH has become increasingly popular over the last decade [4,14], the pandemic has drastically propelled this movement but has also removed the element of choice with many having to WFH without sufficient preparation or facilities. Therefore, some reported challenges of WFH during lockdown may reflect the other conditions brought about by the pandemic such as social distancing, rather than simply WFH itself. This idea is backed by research which found that individuals who were not able to WFH pre-pandemic reported higher stress [11]. Consequently, suggestions for precautionary strategies and countermeasures are different when WFH is a requirement compared to when it is a choice taken with adequate preparation.

The pandemic has changed the way we interact with technology, with evidence of people increasing their use of digital media during lockdown, in some instances to the point of problematic use and addiction [5,6,7,15,16]. There are no agreed upon definitions for internet addiction or PIU. However, PIU is described in the literature as a heterogeneous construct and condition which involves extreme and poorly regulated behaviors regarding the use of the internet which leads individuals to distress and challenges in managing their life in physical world [17,18]. In Al-mourad et al. [19] digital addiction is defined using two constructs. The first relates to the style of use (e.g., being excessive and obsessive). The second relates to the harm associated with that (e.g., mental health and familial relationships). It is also worth noting that PIU is currently not recognized in the Diagnostic and Statistical Manual of Mental Disorder, fifth edition (DSM-5) [20] despite consensus over concerns related to internet overuse, although ‘internet gaming disorder’ has been included in the DSM-5 as a subject for further investigation and was formally recognized in the International Classification of Diseases (ICD-11) [21,22,23].

Research exploring psychosocial factors associated with PIU have consistently identified loneliness, and more recently, low life satisfaction to be meaningful predictors [12,24,25,26,27,28]. These findings are relevant to the lockdown context where many individuals reported that feeling lonely during lockdown worsened their wellbeing [9], so perhaps they may look for connection using the only methods available to them such as social media and the internet [29]. Therefore, it could be possible that the increased loneliness and worsened life satisfaction fueled by the social isolation of lockdown, along with the newfound reliance on the internet are creating conditions ripe for increased PIU. In fact, research carried out during the pandemic so far has revealed such a trend [7,30]. PIU can be associated with a decline in an individual’s academic/work performance, physical health and interpersonal relationships [31], therefore exploring whether the relation still holds true in lockdown where the internet is unavoidable and vital is necessary.

Research exploring PIU has also predominantly focused on gender differences with the majority of studies finding that overall, males tend to demonstrate higher PIU than females [32,33,34,35,36]. While several of these studies explored PIU in adolescents and young adults, our study aimed to add to the literature base by examining PIU in the context of adults WFH where using a computer is the primary option for communication and doing work. Although in this context gender is a static factor (as with personality traits), we will not dismiss it from our research focus as there is evidence to suggest that men and women have differently experienced the challenges of lockdown. For example, women have reportedly experienced higher anxiety, loneliness, and depressive symptoms than men [37,38]. As all of these factors have previously shown to be associated with PIU, it may be that gender differences in PIU during lockdown may be different to those during ‘normal’ times. The aim of our research was to replicate previous studies exploring risk factors of problematic internet use (PIU) under the new conditions of WFH during lockdown in the COVID-19 pandemic.

## 2. Hypotheses Development

In Deutrom et al. [39], in which we utilized the same dataset used in this paper, a confirmatory analysis was performed by revisiting the work conducted in the literature on PIU leading to poor cyber security practices. The underlying estimated operational model was based on a survey conducted while people were influenced by lockdown conditions due to COVID-19, as informed by the key works of [24,25,40]. The model explored how loneliness, life satisfaction, and PIU were associated with cyber security behaviors measured across four subfactors: password generation, devices securement, updating, and proactive awareness [41]. However, in the present study we adopted a different approach informed by the literature discussed in the introductory section, focusing instead on PIU alone and its associations with life satisfaction, loneliness and gender. 

While comparing PIU during lockdown to PIU during ‘normal’ pre-pandemic times would have been ideal, due to the unpredictable and unprecedented nature of COVID-19 there was no perfectly matched sample available to do so. As such, our study aimed to provide a benchmark for PIU data in lockdown conditions which could be utilized in future research looking to compare post-lockdown findings. As many companies are moving towards WFH as the ‘new normal’ [42], these findings have potential importance for employers towards maintaining the wellbeing and healthy online behavior of their employees. Therefore, we aimed to explore whether these previously identified risk factors of PIU are still relevant during the pandemic with the following hypotheses: 

When working from home during lockdown conditions:

**Hypothesis** **1** **(H1).** *Life satisfaction is still associated negatively with PIU.*

**Hypothesis** **2** **(H2).** *Loneliness is still associated positively with PIU.*

**Hypothesis** **3** **(H3).** *PIU is still more prevalent in males than in females.*

**Figure 1:**
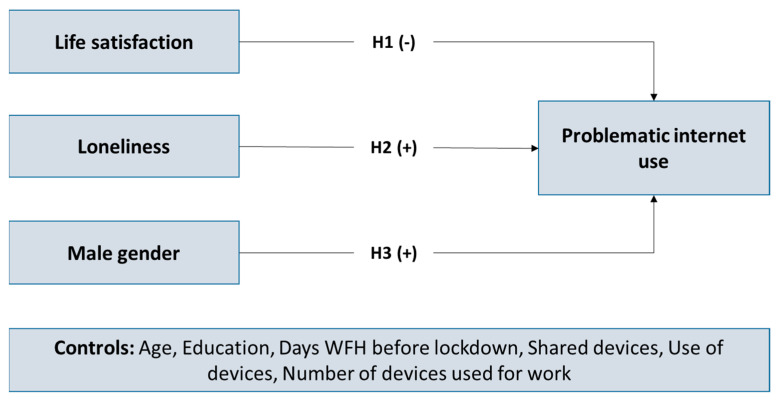
The operational model.

The operational model of the above hypotheses is depicted in Figure 1.

## 3. Methods

### 3.1. Sample 

Recruited via online platform Prolific (https://www.prolific.co/, accessed 16 January 2022), 299 adults WFH in the UK completed the online survey, receiving £1.50 reimbursement for their participation. Participants of all abilities were recruited from all across the UK. Eligibility criteria included being age 18 or over, WFH every day and only having WFH rarely (<one day a week), or sometimes (one day or more) before the pandemic. Individuals who commuted to work, always WFH even before the pandemic or were unemployed were excluded from participating. Pre-screening filters implemented by Prolific ensured that only individuals meeting our criteria were presented with our survey. Ages of participants ranged from 18 to 69 years (*µ*= 33.00, σ = 0.48) and the sample consisted of 183 females (61.20%) and 116 males (38.80%). This study was approved by Bournemouth University ethics process. Data were collected during July 2020 while many people were still WFH as the UK was still in lockdown, although restrictions had started to ease. 

The participant demographics are presented in Table 1 below. 

### 3.2. Measures

We altered the initial instructions for each scale to request that participants consider their experiences of lockdown when completing the questions. However, other than this we presented the scales as they were in their original studies. Table 2 presents details of how each scale and how it was scored. In order to attempt replicating the studies which formed the hypotheses as close as possible, all scales were chosen from the most relevant studies previously discussed [24,36];

Life satisfaction: This first-order construct consists of five items, based on Diener et al.’s Satisfaction with Life Scale (SWLS) [43]. Items include: “In most ways my life is close to my ideal”, “I am satisfied with my life”, “The conditions of my life are excellent”, “If I could live my life over, I would change almost nothing”, and “So far I have gotten the important things I want in life”. This scale was chosen for its brevity and established reliability (α = 0.87).

Loneliness: This first-order construct is composed of three items from the UCLA-3 Item Loneliness Scale (UCLA-3;) [44]. Items include: “How often do you feel isolated from others?”, “How often do you feel left out?” and “How often do you feel that you lack companionship?”. Like the SWLS, this scale was chosen for its brevity and robust testing (α = 0.72).

Problematic internet use: The Problematic Internet Use Questionnaire Short-Form 6 (PIUQ-SF-6;) [45], is a second-order construct consisted of six items, two for each of the three subscales: obsession with the internet use (Cronbach α = 0.76), neglect of other essential everyday needs and jobs (α = 0.59) and control disorder, denoting difficulties in staying in control over Internet use (α = 0.82).

Other: Several demographic questions including items related to WFH environments were presented such as ‘Do you use said device(s) only for work or for personal use (e.g., social media, online shopping, surfing the internet) as well?’ Further demographic information was collected via pre-screening filters and existing data captured by Prolific; these included questions regarding student status, employment status, country of residence and how many days spent WFH. Participants also completed the Security Behavior Intentions Scale (SeBIS;) [41], however that data was not included in the current hypotheses or analysis.

Table 3 contains the descriptive statistics for the psychological measures carried out. When comparing the results to the scoring criteria in Table 2, it can be seen that the mean scores for SWLS, UCLA-3, and PIUQ-SF-6 are all relatively close to their respective neutral points.

### 3.3. Statistical Analysis

Our study utilized a quantitative method by carrying out structural equation modelling (SEM) in AMOS-SPSS, using maximum likelihood estimation methodology. As mentioned in the Hypotheses, the dataset used for this analysis is the same dataset from Deutrom et al. [39]. Content validity of the constructs is accepted, as the literature previously discussed are endorsed for operationalizing these constructs. In this work, we followed a similar approach to assess the model fits (i.e., multiple indices were used as a model can be satisfactory on one fit index but weaker on others) [46]. The following fit indices were used: Chi-square test (with critical significant level *p* > 0.05); the normed-chi-square ratio (critical level of 3 or lower); the normed fit index—NFI (critical level of 0.90 or higher); the goodness of fit index—GFI (critical level of 0.80 or higher); the comparative fit index—CFI (critical level of 0.90 or higher); the standardised root mean squared residuals—SRMR (critical level of 0.08 or lower); and the root mean squared error of approximation—RMSEA (critical level of 0.08 or lower;) [47]. Data from participants with notably short survey completion times were discarded in order to ensure higher validity.

## 4. Results

### 4.1. Data Properties

Table 4 presents the means, the standard deviations, skewness/kurtosis, and the Cronbach alphas for all constructs in the present sample. Also presented are the average variance extracted (AVE) for each construct. These were created by implementing confirmatory factor analysis (CFA), and the bivariate correlation coefficients between all constructs from the study. As all Cronbach alphas are much higher than 0.70, this suggests that construct internal consistency is supported [48]. The scores of AVE for all constructs are higher than 0.50, and therefore construct validity is also supported. Considering each factor’s AVE square root is greater than its correlations with other factors, we can infer that construct discriminant validity is also supported [49]. Moreover, the values of skewness and kurtosis of the constructs in Table 4 range between −1 and +1, verifying that the constructs used in the study follow the normal distribution, allowing thus the use of maximum likelihood estimation methodology [50].

### 4.2. The Measurement Model

Table 4 demonstrates that significantly negative correlation coefficient between life satisfaction and problematic internet use (r = −0.189), and is significantly positive between loneliness and problematic internet use (r = 0.307), supporting our hypotheses. However, due to the exchanges between many variables, although results of correlation coefficients may be interesting, they may ultimately prove perplexing. Therefore, to isolate potential associations between the constructs from the operational model demonstrated in Figure 1, we continued to estimate and examine the measurement and structural models.

By applying CFA to the measurement model which is composed of the constructs presented in Figure 1, the fit indices (Chi-Square = 62.774, df = 41, *p* = 0.000, Normed-Chi-Square = 1.531, GFI = 0.966, CFI = 0.986, NFI = 0.961, SRMR = 0.0345, RMSEA = 0.042) signified sufficient fit. After applying CFA to a model with all items loading on a single factor, the fit indices (Chi-Square = 664.54, df = 77, *p* = 0.000, Normed-Chi-Square = 8.63 GFI = 0.421, CFI = 0.618, NFI = 0.592, SRMR = 0.1261, RMSEA = 0.160) demonstrated to be worse than the hypothesized model, therefore upholding the constructs of the measurement model. When comparing the results of the single factor model and the measurement model (i.e., ratio = Δchi-square/Δdf = 601.766/36), we can deduce that the constructs of our study are sufficiently distinct, and as the ratio of the common method bias (CMB) = 16.7 is much larger that the critical value of 3.84 per degree of freedom we can argue that the CMB is limited [51].

### 4.3. The Structural Model and Testing of Hypotheses

**Figure 2:**
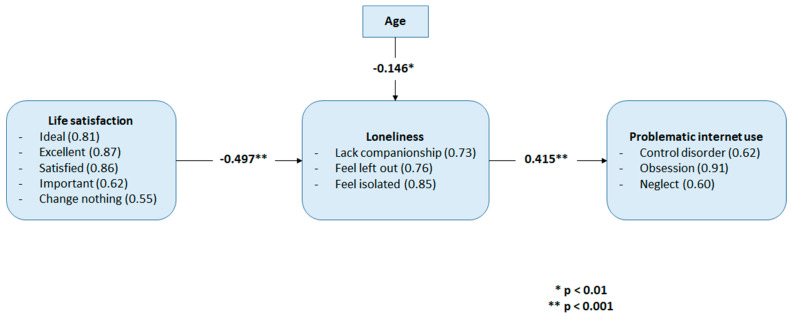
The estimated operational model.

We applied SEM to estimate the model in Figure 1, the fit indices resulted (Chi-Square = 63.206, df = 43, *p* = 0.000, Normed-Chi-Square = 1.470, GFI = 0.966, CFI = 0.987, NFI = 0.960, SRMR = 0.1028, RMSEA = 0.040) indicated a good fit. However, the relationships between satisfaction with life and PIU, and between gender and PIU were not significant, answering H1 and H3, and so were removed from the final model. Furthermore, while there was no significant relationship between life satisfaction and PIU, indirect effects through loneliness were tested. Figure 2 demonstrates estimation results from the control variables, and it must be noted here that only age is present as all other control variables were not significant. The figures in Figure 2 refer to standardized coefficients. Figure 2 shows the significant levels of the structural coefficients, whilst all of the rest estimated coefficients are significant at level *p* = 0.001. The fit indices of the final model produced (Chi-Square = 79.461, df = 49, Normed-Chi-Square = 1.622, GFI = 0.958, CFI = 0.980, NFI = 0.951, SRMR = 0.0464, RMSEA = 0.046) indicated a very good fit. Subsequently, the estimated operational model shown in Figure 2 is considered acceptable.

The results demonstrated in Figure 2 suggest a negative association between life satisfaction and loneliness (β = −0.497, *p* < 0.001), and loneliness is positively associated with PIU (β = 0.415, *p* < 0.001), supporting H2. As there is no direct significant association between life satisfaction and PIU, we can infer that the relationship between life satisfaction and PIU is completely mediated by loneliness. Regarding the control variables, the negative standardized coefficient of age (β = −0.146, *p* < 0.01) in association with loneliness suggests that older individuals in our WFH sample may feel less lonely. Moreover, life satisfaction maintains a negative relation with PIU as observed by the negative sign when multiplying the two relations of life satisfaction—loneliness and loneliness—PIU (−0.497 × 0.415 = −0.2062).

## 5. Discussion

Our study aimed to provide benchmark data for PIU among people WFH during the COVID-19 pandemic lockdown, and to provide new insight on the risk factors associated with PIU. We utilized the same dataset from our previous research [39]. In the present research, we adopted a different approach by conducting an analysis initially focusing on the interplay between life satisfaction, loneliness and gender with PIU. However, through exploratory analysis we examined whether loneliness is a mediating factor between life satisfaction and PIU. We argue that a lockdown may exacerbate the feeling of loneliness which in essence may become a pivotal factor leading to PIU.

In contrast to previous findings [13,24], life satisfaction did not appear to be significantly associated with PIU (answering H1). Loneliness did appear to be positively associated with PIU (answering H2) which supported previous findings [11,12,24]. It should be noted that our results are in agreement with the model in Deutrom et al. [39] where loneliness maintains a positive relation with PIU. While there was no direct relationship between life satisfaction and PIU, life satisfaction was found to be negatively associated with loneliness, suggesting that loneliness fully mediated the relationship between life satisfaction and PIU (i.e., only when individuals are lonely does their life satisfaction influence PIU). Unlike the majority of previous findings [32], our study did not find significant gender differences in PIU (answering H3). Exploration of demographic variables found age to negatively predict loneliness, and although this was not explored in-depth in the original hypothesis it is consistent with previous literature which suggests that younger people reportedly experience more loneliness than middle-aged and older people [52,53]. Again, while age was not significantly associated with PIU directly, it appears that loneliness fully mediated the relationship suggesting that only when individuals are lonely does their age predict PIU. Caution must be made when interpreting this finding in a wider setting as our sample consisted of individuals WFH with only one participant aged over 60. Therefore, our findings are more applicable only to those of typical working age rather than elderly individuals of the overall population. 

The non-significant relationship between life satisfaction and PIU suggests that perhaps life satisfaction is not as important as previously thought, or simply is not as directly influential on PIU during lockdown conditions. However, the significant negative association between life satisfaction and loneliness suggests that life satisfaction still plays a role indirectly. As well as objective factors such as health and economic status, life satisfaction also depends on subjective factors such as meaningful social interaction—something which has been greatly limited in lockdown [54]. Consequently, if those social needs are not fulfilled to the level desired by the individual, they may feel lonely. The significant positive relationship between loneliness and PIU supports the notion that individuals may be more likely to use the internet excessively and problematically in an attempt to feel more socially connected [55,56]. While in the past during pre-pandemic times an over-reliance on technology despite having more options to socialize was associated with loneliness, our current finding suggests that even when people have no other options to socialize during lockdown, their perception of technology did not change. We would expect that technology is a medium to break through loneliness during lockdown and that the negative correlation typically found in normal times shall not apply during lockdown. This finding could be explained by the Social Compensation and Social Enhancement hypotheses. The former predicts that people use the internet to compensate for deficits in offline social support, while the latter posits that people who perceive themselves as having flourishing offline networks use the internet to add to their offline communication [57]. While there is evidence to support each hypothesis in various contexts of interpersonal communication, it is feasible that lockdown may have created a shift towards social enhancement where people attempt to maintain their friendships online. 

Contrasting previous literature which found males to generally exhibit higher PIU, we found no gender differences in PIU during lockdown conditions. There are several possible interpretations of this finding. If our findings are truly reflective of the real world, they suggest that the gender differences in PIU seen during ‘normal’ times are simply not present under lockdown conditions. When looking into the factors contributing to previously reported gender differences, it appears that socio-economic factors such as lower income (GDP) per capita and internet access in a country led to a larger gender-related differences in PIU estimates [32]. Similarly, evidence suggested that the PIU gender gap was higher in countries such as India where social and cultural norms about women and their role may restrict the use of the internet by females, thereby protecting them from developing PIU. Considering that our data were collected in the UK where these factors may not be as prevalent (especially in a population WFH who most likely all have constant internet access), this may partially explain the lack of gender-related PIU differences found. Furthermore, the current findings may instead reflect a shift in PIU and internet use in general that was accelerated during lockdown in both genders. Longitudinal research carried out by ONS found that the difference in active internet users between men and women has been closing over the last decade in the UK [58]. If these findings extend from general internet use to PIU, perhaps upcoming research will see more results such as ours. 

### 5.1. Implications 

Given that PIU has shown to have negative consequences on individuals’ work performance, employers may benefit from applying our findings by monitoring employee wellbeing and easing social connectivity to reduce the chance of developing PIU whilst WFH [59]. Practical recommendations to reduce loneliness and PIU may include providing employees with access to resources such as mental health support should they find themselves severely impacted, but also preventative measures such as adapting social activities amongst staff to take place virtually to keep and boost morale. Similarly, encouraging the use of digital wellbeing apps to raise awareness and improve self-regulation, and even nontechnical deterrence methods such as organizational acceptable use policies to monitor employee web activity may prove useful [7,60]. While loneliness is too complex an issue to completely eliminate using these methods, providing individuals with adequate support and tools is still a step in the right direction to help them manage their internet behaviors. 

While previous studies have also found loneliness to be more prevalent in younger people, the implications of such a result in the context of WFH are less explored [61]. According to ONS, the youngest working age group (aged 16–24) are the least likely to be WFH, however age should not be neglected, as there is evidence to suggest that creating an enriching work environment for younger employees is important in instilling company loyalty and reducing staff turnover [4,62]. Therefore, it still may be of interest to employers to pay attention to PIU in younger employees if this is a demographic which they wish to invest in. 

Despite finding no direct effects of life satisfaction or gender on PIU, our findings here still have useful implications by challenging the existing literature base and providing an insight into PIU specifically in the context of WFH during a pandemic. Employers seeking to minimize PIU among their workers may pay more attention to male employees due to the prevalence of research focused on gender differences, whilst in reality those findings may not be as applicable to them compared to research such as ours, which suggests that loneliness and age are the risk factors which should be prioritized in the WFH context. 

### 5.2. Limitations 

The current study was mainly limited by the measurement of PIU as whole rather than its nuanced manifestations. Some studies focusing more in-depth on the role of gender in PIU looked at the different types of internet activities being carried out, revealing gender differences in specific PIU such as males showing more PIU related to online gaming and gambling whilst women more so with social media and communication [63,64,65]. Unlike these studies, we only utilized the PIUQ-SF-6 which does not compare specific types of PIU but rather measures PIU in general. Consequently, it could be the case that while there were no gender differences in overall PIU, there may have been gender-based differences in the specific types of PIU which our methods were not sensitive to. Similarly, the use of the PIUQ-SF-6 alone meant that only self-reported data was captured, with no data on participants’ actual internet usage. It may be the case that actual levels of PIU varied differently across the factors we examined without being reported. For example, participants’ actual internet usage may be objectively more problematic than during the pre-pandemic period with more hours spent online. However, their perception of what counts as problematic may have warped during lockdown with excessive internet use being justified as a coping strategy rather than maladaptive [66,67,68]. However, this does not detract from the validity of our findings, as regardless of actual differences in internet usage, self-reported data still gives insight of perceived PIU which may be just as impactful on individuals’ wellbeing. 

Furthermore, the relationship between PIU and loneliness is complex. In fact, some evidence suggests that controlling for other variables such as depression reveals a weaker link, and although the PIUQ-SF-6 is multifaceted, when using scales we can only examine what said scales are comprised of, i.e., the subscales and the ideologies behind them [17,69]. For example, the PIUQ-SF-6 is theoretically based on pathological gaming and measures PIU through the dimensions of obsession, neglect, and control disorder, contrasting to other scales such as the Online Cognition Scale which is based on cognitive-behavioral theory and encompasses four dimensions of diminished impulse control, social comfort, loneliness/depression, and distraction [70,71]. While there is some overlap in dimensions, it is inevitable that some aspects of PIU would not be covered if using just one scale.

Finally, our study was limited by the gender imbalance of the sample with a divide of approximately 60% females and 40% males. While this was especially not ideal for research initially exploring gender differences of PIU, the imbalance itself was not statistically significant and as the findings regarding gender were directed towards the null, the risk of bias was limited [72].

### 5.3. Future Directions

Future research could further scrutinize the relationship between gender and PIU among those WFH using more activity-specific and practical methodologies to examine whether our findings are replicated. Furthermore, future research may use our findings as a benchmark to compare results across time as the pandemic progresses to gain a rich, longitudinal insight to PIU and WFH.

## 6. Conclusions

The present study investigated the psychosocial and demographic factors contributing to PIU during the UK COVID-19 lockdown. Our study provided a robust SEM in which loneliness was found to be positively associated with PIU and age negatively associated, supporting previous literature on this subject. While there was no direct effect of life satisfaction on PIU, life satisfaction was negatively associated with loneliness, suggesting that loneliness fully mediated the relationship between life satisfaction and PIU. Conversely, gender showed no significance in predicting PIU, which challenges previous works in the literature and provides new insight into the risk factors of PIU in the context of WFH during a pandemic. Our findings have implications for employers seeking to reduce PIU among their staff WFH, which is becoming increasingly relevant as many companies shift towards remote working permanently. Finally, the contributions of our data as a benchmark of PIU during lockdown conditions may be valuable for those seeking to explore longitudinal patterns of PIU in future waves of COVID-19 and lockdowns as the pandemic progresses and beyond into the ‘new normal’.

## Figures and Tables

**Table 1:** Demographics of the participants.

(*n* = 299)	Frequency	Percent
**Gender**		
Female	183	61.2
Male	116	38.8
**Age (years)**		
18–24	38	12.7
25–29	78	26.1
30–34	65	21.7
35–39	59	19.7
40–44	29	9.7
45–49	19	6.4
50+	11	3.7
**Education**		
No formal education	2	0.7
GCSEs or equivalent	14	4.7
A-Levels or equivalent	58	19.4
Bachelor’s degree	142	47.5
Master’s degree	61	20.4
PhD	17	5.7
Vocational program	4	1.3
Prefer not to say	1	0.3
**WFH before lockdown**		
Rarely, less than one day a week	205	68.6
Sometimes, 1 or more days a week	94	31.4
**Device shared**		
Yes	23	7.7
No	276	92.3
**Device use**		
Just work	66	22.1
Work and personal	233	77.9
**Number of devices used**		
1	189	63.2
2	77	25.8
3	25	8.4
4	8	2.7

**Table 2:** Measurement and scoring of each scale.

Scale	Likert Measure	Scoring
SWLS	7-point: 1 = Strongly disagree, 7 = Strongly agree	Range of scores: 5–35. 20 = neutral point. 5–9 = extremely dissatisfied with life, 31–35 = extremely satisfied.
UCLA-3	3-point: 1 = Hardly ever, 3 = Often	Range of scores: 3–9. Higher scores indicate higher loneliness, >6 = lonely.
PIUQ-SF-6	5-point: 1 = Never, 5 = Always/almost always	Range of scores: 5–30. Higher scores indicate higher PIU, >15 = problematic.

**Table 3:** Descriptive statistics for psychological measures.

Scale	Mean of Total Score	Standard Deviation
SWLS	21.10	6.37
UCLA-3	5.42	1.88
PIUQ-SF-6	13.70	4.46
Obsession	4.09	1.73
Neglect	4.89	1.76
Control	4.71	1.73

**Table 4:** Data properties.

Constructs	Means (Standard Deviations)	Skewness {Kurtosis}	Cronbach Alphas	Correlation Coefficients		
				Life satisfaction	Loneliness	Problematic Internet use
Life satisfaction	4.22 (1.27)	−0.391 {−0.594}	0.867	(0.634)		
Loneliness	1.81 (0.63)	0.432 {−0.813}	0.822	−0.439 **	(0.666)	
Problematic Internet use	2.28 (0.74)	0.277 {−0.337}	0.812	−0.189 **	0.307 **	(0.518)

Notes: ** Correlations are significant at 0.01 level (2-tailed). Figures in brackets indicate Average Variance Extracted (AVE).

## Data Availability

The datasets used and/or analysed during the current study are available from the corresponding author on reasonable request.

## References

[1] Worldometer (2021). COVID-19 Coronavirus Pandemic. https://www.worldometers.info/coronavirus/.

[2] John T., McGee L., Bashir N. (2021). UK Prime Minister Imposes Harsh Lockdown as New COVID-19 Variant Spreads. https://edition.cnn.com/2021/01/04/uk/uk-lockdown-covid-19-boris-johnson-intl/index.html.

[3] Express and Star A Timeline of UK Lockdown Measures Since the Pandemic Began. https://www.expressandstar.com/news/uk-news/2021/01/04/a-timeline-of-uk-lockdown-measures-since-the-pandemic-began/.

[4] Office for National Statistics Coronavirus and Homeworking in the UK: April 2020. https://www.ons.gov.uk/employmentandlabourmarket/peopleinwork/employmentandemployeetypes/bulletins/coronavirusandhomeworkingintheuk/april2020.

[5] Feldmann A., Gasser O., Lichtblau F., Pujol E., Poese I., Dietzel C., Wagner D., Wichtlhuber M., Tapiador J., Vallina-Rodriguez N. (2021). Implications of the COVID-19 Pandemic on the Internet Traffic. Proceedings of the 15th ITG-Symposium.

[6] Reglitz M. (2020). Internet Access Is a Necessity Not a Luxury—It Should Be a Basic Right. https://www.birmingham.ac.uk/schools/ptr/departments/philosophy/news/2020/reglitz-internet-access.aspx.

[7] Király O., Potenza M.N., Stein D.J., King D.L., Hodgins D.C., Saunders J.B., Griffiths M.D., Gjoneska B., Billieux J., Brand M. (2020). Preventing problematic internet use during the COVID-19 pandemic: Consensus guidance. Compr. Psychiatry.

[8] Marshall L., Bibby J., Abbs I. (2020). Emerging Evidence on COVID-19’s Impact on. https://www.health.org.uk/news-and-comment/blogs/emerging-evidence-on-covid-19s-impact-on-mental-health-and-health.

[9] Office for National Statistics Coronavirus and Loneliness, Great Britain: 3 April to 3 May 2020. https://www.ons.gov.uk/peoplepopulationandcommunity/wellbeing/bulletins/coronavirusandlonelinessgreatbritain/3aprilto3may2020.

[10] Zhang S.X., Wang Y., Rauch A., Wei F. (2020). Unprecedented disruption of lives and work: Health, distress and life satisfaction of working adults in China one month into the COVID-19 outbreak. Psychiatry Res..

[11] Costa R.M., Patrão I., Machado M. (2019). Problematic internet use and feelings of loneliness. Int. J. Psychiatry Clin. Pract..

[12] Kim J., LaRose R., Peng W. (2009). Loneliness as the Cause and the Effect of Problematic Internet Use: The Relationship between Internet Use and Psychological Well-Being. CyberPsychol. Behav..

[13] Kabasakal Z. (2015). Life satisfaction and family functions as-predictors of problematic Internet use in university students. Comput. Hum. Behav..

[14] Ceyhan A.A., Ceyhan E. (2008). Loneliness, Depression, and Computer Self-Efficacy as Predictors of Problematic Internet Use. Cyber Psychol. Behav..

[15] Garfin D.R. (2020). Technology as a coping tool during the coronavirus disease 2019 (COVID-19) pandemic: Implications and recommendations. Stress Health.

[16] Cellini N., Canale N., Mioni G., Costa S. (2020). Changes in sleep pattern, sense of time and digital media use during COVID-19 lockdown in Italy. J. Sleep Res..

[17] Moretta T., Buodo G. (2020). Problematic Internet Use and Loneliness: How Complex Is the Relationship?. A Short Literature Review. Curr. Addict. Rep..

[18] Caplan S.E. (2005). Refining the Cognitive Behavioral Model of Problematic Internet Use.

[19] Almourad M.B., McAlaney J., Skinner T., Pleya M., Ali R. (2020). Defining digital addiction: Key features from the literature. Psihologija.

[20] American Psychiatric Association (2013). Diagnostic and Statistical Manual of Mental Disorders.

[21] Cho H., Kwon M., Choi J.-H., Lee S.-K., Choi J.S., Choi S.-W., Kim D.-J. (2014). Development of the Internet addiction scale based on the Internet Gaming Disorder criteria suggested in DSM-5. Addict. Behav..

[22] Jeong E.J., Kim D.J., Lee D.M. (2016). Why Do Some People Become Addicted to Digital Games More Easily? A Study of Digital Game Addiction from a Psychosocial Health Perspective. Int. J. Hum. Comput. Interact..

[23] World Health Organization (2018). Inclusion of “Gaming Disorder” in ICD-11. https://www.who.int/news/item/14-09-2018-inclusion-of-gaming-disorder-in-icd-11.

[24] Shinkins A. (2016). Examination of the Relationship between Online Cognition, Predictor Variables of Psychosocial Well-Being and Personality Traits. Higher Diploma in Arts in Psychology.

[25] Dhir A., Chen S., Nieminen M. (2015). A repeat cross-sectional analysis of the psychometric properties of the Compulsive Internet Use Scale (CIUS) with adolescents from public and private schools. Comput. Educ..

[26] Celik V., Yesilyurt E. (2013). Attitudes to technology, perceived computer self-efficacy and computer anxiety as predictors of computer supported education. Comput. Educ..

[27] Bhagat S., Jeong E.J., Kim D.J. (2020). The Role of Individuals’ Need for Online Social Interactions and Interpersonal Incompetence in Digital Game Addiction. Int. J. Hum. Comput. Interact..

[28] Leite Â., Ramires A., Amorim S., Sousa H.F.P.E., Vidal D.G., Dinis M.A.P. (2020). Psychopathological Symptoms and Loneliness in Adult Internet Users: A Contemporary Public Health Concern. Int. J. Environ. Res. Public Health.

[29] "Irani T.A., Wilson S.B., Slough D.L., Rieger M. (2014). Graduate Student Experiences on-and off-Campus: Social Connectedness and Perceived Isolation. Int. J. E-Learn. Distance Educ..

[30] Alheneidi H., AlSumait L., AlSumait D., Smith A.P. (2021). Loneliness and Problematic Internet Use during COVID-19 Lock-Down. Behav. Sci..

[31] Shek D.T.L., Sun R.C.F., Yu L., Pfaff D.W., Martin E., Pariser E. (2013). Internet Addiction.

[32] Su W., Han X., Jin C., Yan Y., Potenza M.N. (2019). Are males more likely to be addicted to the internet than females? A meta-analysis involving 34 global jurisdictions. Comput. Hum. Behav..

[33] Tomaszek K., Muchacka-Cymerman A. (2019). Sex Differences in the Relationship between Student School Burnout and Problematic Internet Use among Adolescents. Int. J. Environ. Res. Public Health.

[34] Baloğlu M., Şahin R., Arpaci I. (2020). A review of recent research in problematic internet use: Gender and cultural differences. Curr. Opin. Psychol..

[35] Bulut Serin N. (2011). An Examination of Predictor Variables for Problematic Internet Use. Turk. Online J. Educ. Technol. TOJET.

[36] Opakunle T., Aloba O., Opakunle O., Eegunranti B. (2020). Problematic Internet Use Questionnaire-Short Form-6 (PIUQ-SF-6): Dimensionality, validity, reliability, measurement invariance and mean differences across genders and age categories among Nigerian adolescents. Int. J. Ment. Health.

[37] Bu F., Steptoe A., Fancourt D. (2020). Loneliness during a strict lockdown: Trajectories and predictors during the COVID-19 pandemic in 38,217 United Kingdom adults. Soc. Sci. Med..

[38] Pieh C., Budimir S., Probst T. (2020). The effect of age, gender, income, work, and physical activity on mental health during coronavirus disease (COVID-19) lockdown in Austria. J. Psychosom. Res..

[39] Deutrom J., Katos V., Ali R. (2021). Loneliness, life satisfaction, problematic internet use and security behaviours: Re-examining the relationships when working from home during COVID-19. Behav. Inf. Technol..

[40] Hadlington L. (2017). Human factors in cybersecurity; examining the link between Internet addiction, impulsivity, attitudes towards cybersecurity, and risky cybersecurity behaviours. Heliyon.

[41] Egelman S., Peer E. Scaling the security wall: Developing a security behavior intentions scale (sebis). Proceedings of the 33rd Annual ACM Conference on Human Factors in Computing Systems.

[42] Felstead A., Henseke G. (2017). Assessing the growth of remote working and its consequences for effort, well-being and work-life balance. New Technol. Work Employ..

[43] Diener E.D., Emmons R.A., Larsen R.J., Griffin S. (1985). The satisfaction with life scale. J. Personal. Assess..

[44] Hughes M.E., Waite L.J., Hawkley L.C., Cacioppo J.T. (2004). A short scale for measuring loneliness in large surveys: Results from two population-based studies. Res. Aging.

[45] Demetrovics Z., Király O., Koronczai B., Griffiths M., Nagygyörgy K., Elekes Z., Tamás D., Kun B., Kökönyei G., Urbán R. (2016). Psychometric Properties of the Problematic Internet Use Questionnaire Short-Form (PIUQ-SF-6) in a Nationally Representative Sample of Adolescents. PLoS ONE.

[46] Bollen K.A. (1989). Measurement models: The relation between latent and observed variables. Struct. Equ. Latent Var..

[47] Hu L.-T., Bentler P.M. (1999). Cutoff criteria for fit indexes in covariance structure analysis: Conventional criteria versus new alternatives. Struct. Equ. Modeling Multidiscip. J..

[48] Nunnally J.C. (1994). Psychometric Theory 3E.

[49] Hair J., Black W., Babin B.Y.A., Anderson R., Tatham R. (2010). Multivariate Data Analysis.

[50] Byrne B.M. (2012). Structural Equation Modeling with Mplus: Basic Concepts, Applications and Programming.

[51] Schaller T.K., Patil A., Malhotra N.K. (2015). Alternative techniques for assessing common method variance: An analysis of the theory of planned behavior research. Organ. Res. Methods.

[52] Barreto M., Victor C., Hammond C., Eccles A., Richins M.T., Qualter P. (2021). Loneliness around the world: Age, gender, and cultural differences in loneliness. Pers. Individ. Differ..

[53] Office for National Statistics (2018). Loneliness—What Characteristics and Circumstances are Associated with Feeling Lonely?. https://www.ons.gov.uk/peoplepopulationandcommunity/wellbeing/articles/lonelinesswhatcharacteristicsandcircumstancesareassociatedwithfeelinglonely/2018-04-10.

[54] Tian Y., Zhang S., Wu R., Wang P., Gao F., Chen Y. (2018). Association Between Specific Internet Activities and Life Satisfaction: The Mediating Effects of Loneliness and Depression. Front. Psychol..

[55] Hardie E., Tee M.Y. (2007). Excessive Internet use: The role of personality, loneliness and social support networks in Internet Addiction. Aust. J. Emerg. Technol. Soc..

[56] McIntyre E., Wiener K.K., Saliba A.J. (2015). Compulsive Internet use and relations between social connectedness, and introversion. Comput. Hum. Behav..

[57] Ruppel E.K., McKinley C.J. (2015). Social Support and Social Anxiety in Use and Perceptions of Online Mental Health Resources: Exploring Social Compensation and Enhancement. Cyberpsychol. Behav. Soc. Netw..

[58] Office for National Statistics Internet Users, UK: 2019. https://www.ons.gov.uk/businessindustryandtrade/itandinternetindustry/bulletins/internetusers/2019#main-points.

[59] Greenfield D.N., Davis R.A. (2002). Lost in Cyberspace: The Web @ Work. CyberPsychol. Behav..

[60] Shepherd M.M., Mejias R.J. (2016). Nontechnical Deterrence Effects of Mild and Severe Internet Use Policy Reminders in Reducing Employee Internet Abuse. Int. J. Hum. Comput. Interact..

[61] Laconi S., Tricard N., Chabrol H. (2015). Differences between specific and generalized problematic Internet uses according to gender, age, time spent online and psychopathological symptoms. Comput. Hum. Behav..

[62] Trapero F.G.A., Castano L.E.V., Parra J.C.V., Garcia J.D.L.G. (2017). Differences on self-perception of organizational pride and loyalty in Millennial & Generation X, considering gender and seniority variables. Bus. Econ. Horiz. (BEH).

[63] Dufour M., Brunelle N., Tremblay J., Leclerc D., Cousineau M.-M., Khazaal Y., Légaré A.-A., Rousseau M., Berbiche D. (2016). Gender Difference in Internet Use and Internet Problems among Quebec High School Students. Can. J. Psychiatry.

[64] Ifdil I., Putri Y.E., Fadli R.P., Erwinda L., Suranata K., Ardi Z., Fitria L., Churnia E., Zola N., Barriyah K. (2018). Measuring internet addiction: Comparative studies based on gender using Bayesian analysis. J. Physics: Conf. Ser..

[65] Su W., Han X., Yu H., Wu Y., Potenza M.N. (2020). Do men become addicted to internet gaming and women to social media? A meta-analysis examining gender-related differences in specific internet addiction. Comput. Hum. Behav..

[66] Nimrod G. (2020). Changes in Internet Use When Coping with Stress: Older Adults During the COVID-19 Pandemic. Am. J. Geriatr. Psychiatry.

[67] Sharma M.K., Thakur P.C., Anand N., Mondal I., Singh P., Ajith S., Kande J.S., Venkateshan S. (2020). Internet use: A boon or a bane during COVID-19. J. Ment. Health Hum. Behav..

[68] Cauberghe V., Van Wesenbeeck I., De Jans S., Hudders L., Ponnet K. (2021). How Adolescents Use Social Media to Cope with Feelings of Loneliness and Anxiety During COVID-19 Lockdown. Cyberpsychol. Behav. Soc. Netw..

[69] Lopez-Fernandez O., Kuss D.J. (2020). Preventing Harmful Internet Use-Related Addiction Problems in Europe: A Literature Review and Policy Options. Int. J. Environ. Res. Public Health.

[70] Davis R.A., Flett G.L., Besser A. (2002). Validation of a New Scale for Measuring Problematic Internet Use: Implications for Pre-employment Screening. CyberPsychol. Behav..

[71] Laconi S., Rodgers R.F., Chabrol H. (2014). The measurement of Internet addiction: A critical review of existing scales and their psychometric properties. Comput. Hum. Behav..

[72] Chasan-Taber L. (2014). Writing Dissertation and Grant Proposals: Epidemiology, Preventive Medicine and Biostatistics.

